# Membrane resistance and shunting inhibition: where biophysics meets state‐dependent human neurophysiology

**DOI:** 10.1113/JP271452

**Published:** 2016-05-12

**Authors:** Walter Paulus, John C. Rothwell

**Affiliations:** ^1^ Department of Clinical NeurophysiologyUniversity of Göttingen Medical CentreGermany; ^2^ UCL Institute of NeurologyQueen SquareLondonWC1N 3BGUK

## Abstract

Activation of neurons not only changes their membrane potential and firing rate but as a secondary action reduces membrane resistance. This loss of resistance, or increase of conductance, may be of central importance in non‐invasive magnetic or electric stimulation of the human brain since electrical fields cause larger changes in transmembrane voltage in resting neurons with low membrane conductances than in active neurons with high conductance. This may explain why both the immediate effects and after‐effects of brain stimulation are smaller or even reversed during voluntary activity compared with rest. Membrane conductance is also increased during shunting inhibition, which accompanies the classic GABA_A_ IPSP. This short‐circuits nearby EPSPs and is suggested here to contribute to the magnitude and time course of short‐interval intracortical inhibition and intracortical facilitation.

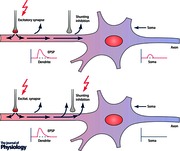

**Abbreviations**
ICF: intracortical facilitation
MEP: motor‐evoked potential
rTMS: repetitive transcranial magnetic stimulation
SICI: short‐interval intracortical inhibition
SICF: short‐interval intracortical facilitation
tACS: transcranial alternating current stimulation
tDCS: transcranial direct current stimulation
TBS: theta burst stimulation
TMS: transcranial magnetic stimulation
TS: transcranial stimulation

## Introduction

A variety of different transcranial stimulation (TS) techniques are now available to modulate brain excitability and explore neuroplasticity in the human brain. They range from brain polarization with transcranial direct current stimulation (tDCS) (Nitsche & Paulus, 2000) to transcranial alternating current stimulation (tACS) (Antal *et al*. 2008) and short‐duration current pulses of about 100 μs produced by transcranial magnetic stimulation (TMS). tDCS and tACS apply small constant or alternating currents (usually 1–2 mA for several minutes) via electrodes placed on the scalp. This is thought to polarize the membranes of cortical neurons by a small amount (< 1 mV). By exerting a constant de‐ or hyper‐polarization, tDCS will tend to increase or decrease the ongoing discharge rate. TACS will do the same in an alternating fashion, and, by acting on a population of neurons over a large area, could potentially interact with ongoing rhythmic activity in cortical networks. The current intensity induced by TMS is much greater and is designed to activate axons and directly initiate action potentials in brain circuits. It produces a highly synchronized bout of neural activity followed by a long period of GABA‐mediated intracortical inhibition. Thus TMS evokes additional activity whereas tACS can synchronize ongoing activity (Ali *et al*. 2013) and tDCS modulates overall levels of ongoing activity (Bindman *et al*. 1964).

All methods can lead to long‐lasting changes in excitability that outlast the stimulation by several minutes up to hours or even days. Here we consider only tDCS and TMS since they have been the most intensively studied. In order to achieve long‐lasting effects, tDCS has to be applied continuously at least for several minutes (Nitsche & Paulus, 2000) whereas TMS has to be applied repetitively (repetitive transcranial magnetic stimulation; rTMS) (Quartarone *et al*. 2006). Low frequencies of rTMS (a typical protocol would use 1800 stimuli applied at 1 Hz) tend to reduce whereas high frequencies (10 or 20 Hz) usually increase excitability; other forms of rTMS such as theta burst stimulation (TBS) can produce both types of effect depending on the pattern of the stimulus pulses (Huang *et al*. 2005; Hamada *et al*. 2008; for an overview see Ziemann *et al*. 2008). Although tDCS has a long history, dating back to the time soon after the development of the voltaic pile (Hellwag & Jacobi, 1802) its long‐lasting after effects only became clear when it became possible to measure them objectively by comparing TMS‐evoked motor‐evoked potentials (MEPs) before and after tDCS (Nitsche & Paulus, 2000; overview in Nitsche *et al*. 2008). With tDCS, cortical pyramidal cells that are aligned parallel to the applied field (that is, with the anode applied to the brain surface above the cell body and the cathode at a distance), will have their dendrites hyperpolarized and the soma depolarized (Rahman *et al*. 2013). The effect of this in the hand area of motor cortex is that application of the anode over M1 increases corticospinal excitability whereas it is reduced with cathodal stimulation (Nitsche & Paulus, 2000). However, it is important to note that this is an idealized description. In practice the surface of the brain is highly folded and can change the orientation of the pyramidal cells to the electric field. An additional consideration is that tDCS can affect the excitability of synaptic inputs onto the pyramidal neurons, again with the proviso that the electric field is oriented parallel to the presynaptic inputs. Inputs arriving in the direction of the field are enhanced whereas those coming from the opposite direction are suppressed (Kabakov *et al*. 2012). It is possible that detailed modelling of electric fields may be able to describe the likelihood of particular patterns of polarization in a particular area of an individual's brain. At present studies are still ongoing (e.g. Shahid *et al*. 2014).

No rose without a thorn, these simple rules – excitation by either high frequency rTMS or anodal tDCS, inhibition by low‐frequency stimulation or cathodal tDCS (Quartarone *et al*. 2006) – apply only when investigated in relaxed subjects at rest. If applied during activation the results may be considerably different. For example, if tDCS of motor cortex is applied during voluntary muscle activation, anodal tDCS no longer produces excitation; instead, both anodal and cathodal stimulation now induce inhibition as measured by their effects on MEPs (Antal *et al*. 2007). Similarly, the effects of the TBS protocols of TMS are abolished if they are applied during concurrent voluntary contraction (Huang *et al*. 2008). Many factors change between rest and activity. There are changes in firing rates, of recurrent transsynaptic excitation via axon collaterals, of the balance between inhibitory and facilitatory synaptic neurotransmission, and in the spectrum and magnitude of oscillatory activity. The excitability of connections to and from the stimulated site will change and influence firing rates within distributed networks. All these things and more will affect the physiological and behavioural reaction to tDCS and rTMS.

Here we focus on one simple factor that we believe to have been underestimated in the past. We ask whether the change in membrane resistance that occurs during activation could contribute to changes in the response to brain stimulation as compared with rest. We will also highlight the role of ‘shunting inhibition’, defined as an increase in synaptic conductance in the absence of an obvious change in membrane potential that can short‐circuit currents generated at adjacent synapses. An enhanced membrane conductance attenuates the membrane depolarization induced by a given current and reduces its effect on voltage‐gated channels in the postsynaptic membrane (Destexhe *et al*. 2003).

## Membrane conductance and resistance

Intra‐ and extracellular fluids are highly conductive; in contrast a cell's membrane is highly resistive. The voltage gradient between outside and inside of a cell is therefore maintained almost entirely across the narrow width of the membrane itself. EPSPs and IPSPs occur when conductive ion channels in the membrane open after binding a molecule of neurotransmitter. Thus both inhibition and excitation are accompanied by a reduction in postsynaptic membrane resistance (i.e. increased conductance). The opening time of ion channels can be much shorter than the duration of the recorded changes in membrane potential since the latter depends on the time constant of the membrane. Thus for any one channel, the change in resistance is much shorter lasting than the change in membrane voltage. However, during normal physiological activity, many channels are active simultaneously and the net effect is that the total membrane resistance is continuously smaller than during periods of inactivity.

Impedance falls whether the inputs are excitatory or inhibitory. In fact, the opening of membrane ion channels by generalized brain activity can change the bulk conductivity of a brain region, a phenomenon that is used in electrical impedance tomography (Liston *et al*. 2012). Maximal resistivity changes estimated with DC currents decrease by 2.8% in fully depolarized unmyelinated crab nerve and 0.6% (0.06–1.7%) in cerebral cortex when 10% of neurons are active during the peak of an evoked response (Liston *et al*. 2012). At localized regions of the membrane this can lead to a drop in resistance by as much as 70% (Pare *et al*. 1998). The voltage domain of interest is expected to be around ± 10 mV around the resting potential (Koch *et al*. 1990). Conductance changes vary depending on the cell types involved. For the inhibitory basket cell projection onto the cell body of pyramidal cells, a 30% increase in somatic input conductance causes a 70% reduction in the amplitude of excitation (Koch *et al*. 1990).

Here we propose that transmembrane resistance plays a larger role in determining the response to TMS and tDCS than has previously been considered. This is because the transmembrane resistance of a neuron is lower during periods of synaptic activity, and this will reduce the amount that the membrane is polarized by external current. Thus, low resistance will reduce the ability of tDCS to change the membrane potential. The effect of activity on the response to TMS is slightly different. TMS activates axons of neurons that synapse onto corticospinal (and other) neurons. Changes in membrane resistance at the soma and dendrites will not affect saltatory transmission within the axon; thus once spikes are elicited by TMS, their propagation does not depend on membrane resistance in the soma or dendrites. However, their postsynaptic inputs in the target cells will arrive at dendritic and somatic membranes where resistance has changed. The lower the resistance, the smaller the distance over which the EPSP/IPSP will spread away from the synapse. This means that EPSP‐induced current flow from the dendritic to the somatic compartment is reduced by increased ‘leaky’ membrane conductance, which in turn may reduce firing gain (Capaday & van Vreeswijk, 2006).

Although synaptic activation (inhibitory or excitatory) always reduces membrane resistance, it is important to note that membrane resistance can increase if ongoing synaptic input is reduced. Since there is continuous activity in the brain even when at ‘rest’, it is possible that a behaviour could reduce input to some neurons and therefore increase their membrane resistance, potentially meaning that they become more sensitive to remaining inputs. Even with the same area of cortex, different neurons behave differently during rest and activation. In the sensory system somatostatin‐expressing neurons hyperpolarized and reduced action potential firing during both passive and active whisker sensing whereas all other recorded types of nearby neurons were excited by sensory input (Gentet *et al*. 2012).

## State‐dependent effects of tDCS and rTMS

As examples of the possible effect of changes in membrane resistance during activation *versus* rest we consider three examples. Volitional contraction of a muscle increases the level of excitatory inputs to corticospinal neurons in order to depolarize the membrane potential and initiate action potentials. Since the membrane potential is nearer to threshold, TMS pulses will need to recruit smaller amplitude EPSPs to alter the neuronal firing rate. However, the reduced resistance of the membrane will mean that EPSPs evoked by TMS are smaller than at rest. These two opposing factors could potentially cancel each other out. In practice there may be a small overall increase in excitability since direct recordings of descending activity from the epidural space of the cervical cord show (at least in some individuals, during high levels of contraction) that volitional activity reduces the threshold for evoking corticospinal responses and increases the number of I‐waves recruited by suprathreshold TMS pulses (Di Lazzaro *et al*. 1998). Similarly in animal experiments, depolarization during activation makes EPSPs reach threshold faster, leading to faster onset spikes for intracortical responses (Castro‐Alamancos, 2009).

The second example is the abolition of the after‐effects of the theta burst rTMS paradigm (TBS) when it is applied during voluntary activation rather than at rest (Huang & Rothwell, 2004). Changes in synaptic plasticity during periods of increased activity have been described in animal preparations. Spontaneous spiking leads to a decrease in amplitude and efficacy of EPSPs (Urban‐Ciecko *et al*. 2015) as well as change in EPSP short‐term plasticity in response to trains of presynaptic action potentials. When spontaneous activity was absent, EPSPs showed short‐term depression that switched to facilitation in a more active slice (Urban‐Ciecko *et al*. 2015).

In humans, theta burst stimulation is thought to cause influx of Ca^2+^ ions through the NMDA receptor (Huang *et al*. 2011). It is assumed that normally the EPSPs from the individual stimuli within a theta burst summate to depolarize the membrane sufficiently to expel the Mg^2+^ ion block in the NMDA receptor channel. The lower membrane resistance during periods of activity may reduce the time constant of the membrane (Delgado *et al*. 2010), shorten the EPSP duration and reduce the amount of summation that is possible. This would decrease the probability that the Mg^2+^ would be expelled from the NMDA receptor and the response to TBS would be abolished as observed in practice (Huang *et al*. 2008). Indeed, it is well known that induction of LTP is more difficult to achieve in the neocortex of freely moving animals than in brain slices or anaesthetized animals (Trepel & Racine, 1998; Froc *et al*. 2000).

The third example is that long‐lasting effects of tDCS change during a period of volitional activity. Reducing transmembrane resistance will reduce the degree of polarization experienced by neurons during tDCS. This might account for the absence of an after‐effect of motor cortex anodal tDCS when applied during voluntary contraction (Antal *et al*. 2007). In fact, increased conductivity during contraction may also explain why the inhibitory response to cathodal tDCS was increased. Since activity tends to reduce excitability by increasing membrane conductivity, it will complement the hyperpolarization of membranes by cathodal tDCS and further reduce the effectiveness of EPSPs.

This argument may also apply for tACS (Moliadze *et al*. 2010). In the resting state 10 min 140 Hz alternating current stimulation increases cortical excitability, compared with a control condition at 80 Hz. Stimulation at 250 Hz led to a delayed increase in excitability with a shorter after‐effect. If tACS was applied during finger tapping most of the excitatory effects disappeared and were replaced by inhibition for placebo, 80 and 250 Hz stimulation. Excitation remained only after stimulation with 140 Hz, which we interpret as a residual excitability increase caused by the most effective 140 Hz frequency being least affected by reduced membrane resistance.

## Potential implication for guidance of tDCS and rTMS after‐effects

A central question to be answered in TS research is how to stimulate as selectively as possible in order to obtain intended behavioural alterations or improvement of symptoms in disease. Any current flow applied by TS techniques can never be restricted to individual cells: it influences thousands of cell bodies with their dendrites and axonal compartments simultaneously. Thus we have to account not only for individual cell behaviour, but also for cell populations that are operational in a given task. The task of TS would be to enhance or inhibit this task. As noted above, due to increased membrane conductance during performance of a task, TS will be potentially less effective in the activated cell groups. This has interesting implications for the concept of surround inhibition at least in the motor cortex (Beck & Hallett, 2011). If there is less synaptic activity in the ‘surround’ than in the ‘active’ area, TS may be more effective on neurons in the less active cell assemblies of the ‘surround’ than it is on the cells that are active in the task being performed.

### GABA_A_: hyperpolarization, depolarization and shunting inhibition

GABA_A_ receptor activation causes Cl^–^ channels to open in the membrane and the usual consequence is a change in the membrane potential. Since the Cl^–^ equilibrium potential is usually more negative than the resting membrane potential, this gives rise to an influx of Cl^–^ and hyperpolarization of the postsynaptic neuron observed as an IPSP (Glickfeld *et al*. 2009). However, the synaptic equilibrium potential for chloride is not always more negative than the resting potential, particularly in early development, or after a period of sustained Cl^–^ influx sufficient to increase the chloride concentration inside the cell (Staley *et al*. 1995). If the synaptic reversal potential is more positive, lying between the resting potential and the threshold for the generation of action potentials, activation of the GABAergic synapse can depolarize the membrane and produce an EPSP. Finally, if the Cl^–^ equilibrium potential is equal to resting potential, then there will be no obvious PSP after activation of a GABA receptor.

In the adult, activation of the GABA receptor is usually assumed to produce an inhibitory IPSP. However, this is not always the case. Certain forms of epilepsy are associated with higher levels of intracellular Cl^–^ that can potentially convert GABAergic inputs into excitatory EPSPs (Huberfeld *et al*. 2015). In principal cells of the hippocampal dentate gyrus, GABAergic inputs are depolarizing whilst they are hyperpolarizing in the CA1 principal cells (Sauer *et al*. 2012). Cortical pyramidal cells receive GABAergic inputs at the axon initial segment. Because Cl^–^ levels here are higher than in the soma and dendrites, this input can depolarize the membrane and initiate action potentials (Szabadics *et al*. 2006). In fact, some work suggests that the effect depends on ongoing levels of activity. Chandelier cells targeting the initial segment of layer 3 pyramidal neurons have GABAergic synapses that are depolarizing at rest but hyperpolarizing during periods of activation (Woodruff *et al*. 2011).

**Figure 1:**
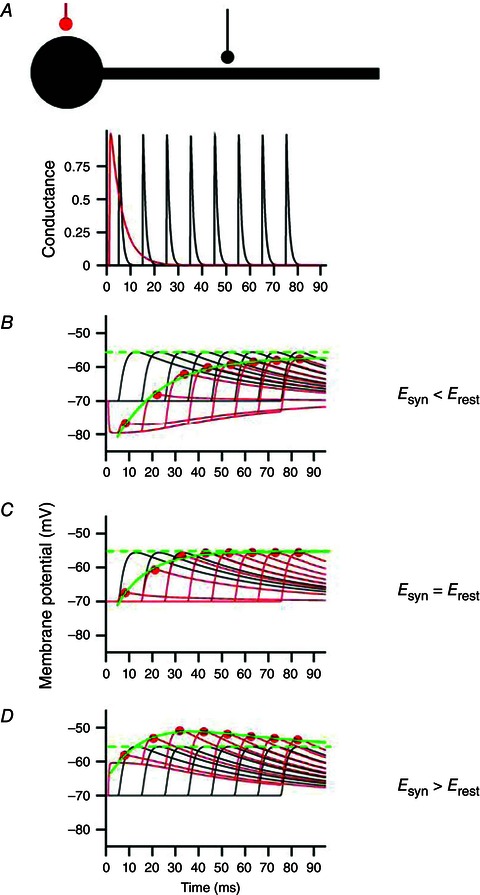
**Different temporal integration rules for hyperpolarizing and shunting inhibition** *A*, top, reduced model of the soma and dendrite of a neuron, with an inhibitory synapse (red) attached to the soma and an excitatory synapse (black) at the dendrite. *A*, bottom, time courses of the postsynaptic conductances generated at the two synapses (the excitatory synapse is activated multiple times). *B*–*D*, integration of inhibitory and excitatory events (red, inhibitory synapse activated; black, inhibitory synapse inactive). Adapted from Jonas & Buzsaki (2007), with permission.

**Figure 2:**
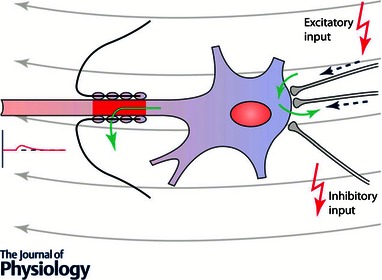
**A cortical pyramidal cell is depicted with two somatic excitatory and one inhibitory synapse** Opening of channels during voluntary activation allows for a cation and anion exchange via the cell's membrane, leading to increased membrane conductance and less efficient rTMS effects. The cell is assumed to be localized in the wall of the precentral gyrus and thus is oriented horizontally (Rathelot & Strick, 2009). Tangential current flow oriented anterior‐posteriorly as induced by TMS is symbolized by grey arrows. Green arrows indicate inward or outward current flow. Since in most cases rTMS is applied at subthreshold levels the axon hill remains subthreshold and does not induce a spike. In case of paired‐pulse stimulation suprathreshold stimulation of S2 will elicit a spike, but S1 will increase membrane conductance and via shunting inhibition reduce the somatic depolarization produced by S2. Modified from Kandel & Siegelbaum (2000).

Opening the chloride channel not only allows free flow of chloride ions across the membrane but also reduces the transmembrane resistance. The result is a dual inhibitory effect on ongoing excitatory inputs: hyperpolarization subtracts from the depolarizing effect of an EPSP while the lower membrane resistance reduces its amplitude. The effect is known as shunting inhibition and is particularly effective in ‘withdrawing’ depolarizing current brought in by nearby excitatory synapses (Furman, 1965). See Box 1 and Figs 1 and 2.

**Box 1: Shunting inhibition in relation to the resting membrane potential**

Depending on the relation between the synaptic reversal potential (SP) for Cl^–^ ions and the resting potential (RP) three different scenarios can be differentiated (Jonas & Buzsaki, 2007). Conceptual explanations of rTMS and tDCS effects depend on the following relations.

(i) (Fig. 1 *C*) SP = RP: shunting inhibition does not produce significant de‐ or hyperpolarizing currents if the Cl^–^ reversal potential is close to the resting membrane potential. No measurable IPSPs or EPSPs are generated. The only effect is due to the increased local membrane conductance, which can shunt excitability changes evoked by other inputs at nearby synapses.
(ii) (Fig. 1 *D*) RP > SP: if the resting potential is less negative than the Cl^–^ equilibrium potential, activation of the inhibitory synapse generates a classic inhibitory postsynaptic potential (IPSP). The time course of this inhibition is longer than shunting inhibition and follows the IPSP. However, it should be noted that during channel opening, shunting inhibition will add to these effects on membrane potential and increase its effectiveness.
(iii) (Fig. 1 *B*) RP < SP: when the resting membrane potential is more negative than the Cl^–^ reversal potential GABAergic stimulation leads to a paradoxical depolarization. However this effect on membrane voltage is counteracted, at least at the start of the PSP, by the concomitant reduction in transmembrane resistance (Gulledge & Stuart, 2003). In the early, shunting phase, conductance is high and can suppress the excitatory effects of nearby EPSPs by shunting their current. In contrast, nearby EPSPs that occur later will sum with the residual depolarization and be facilitated (Gulledge & Stuart, 2003). The difference between resting and chloride reversal potential amounts to about 10 mV in mammalian neocortical pyramidal cells (Gulledge & Stuart, 2003) but usually this is less than action potential threshold, which is more positive than a maximally depolarized *E*_GABA_.

GABA_B_ receptors are located both on presynaptic terminals and on the cell body and dendrites of pyramidal neurons. Presynaptic GABA_B_ receptors cause inhibition of voltage‐sensitive Ca^2+^ channels and reduce transmitter release. Postsynaptically, GABA_B_ receptors can lead to inhibition via activation of G‐protein‐coupled inwardly rectifying potassium channels. These will both hyperpolarize the membrane and increase membrane conductance, a combined action similar to that at the GABA_A_ receptor. However, it has been studied in much less detail and is not considered further here.

### Spatial and temporal properties of shunting inhibition

Shunting inhibition is particularly effective in reducing the effect of more distally located excitatory inputs. This is sometimes referred to as ‘on‐the‐path’ inhibition. Thus, GABAergic synapses on a proximal portion of dendrite will reduce the effectiveness of all distal EPSPs, but have much less effect on EPSPs generated at more proximal synapses (Hao *et al*. 2009). Effectively, shunting inhibition suppresses excitation from distal sources that must pass through that region on the way to the soma, whereas it has less influence on proximal EPSPs that influence the soma directly.

Shunting inhibition can be quantified by the sum of the EPSP, the IPSP and a nonlinear term proportional to their product (*k* × EPSP × IPSP), where the coefficient *k* reflects the strength of shunting effect (Hao *et al*. 2009). The *k *value strongly depends on the locations (dendritic trunk, oblique branches, soma) of excitatory and inhibitory inputs and the distance between them. The duration of shunting inhibition is relatively short in comparison with the membrane polarization that is produced at the GABAergic synapse. This is because transmembrane resistance is reduced while the Cl^–^ channel is open; it returns to normal when the channel closes. The duration of shunting inhibition depends on the duration of IPSPs and EPSPs, which in the cat have been estimated as to have the values shown in Table 1 (Thomson *et al*. 2002).

**Table 1:** Duration of IPSPs and EPSPs in the cat (from Thomson *et al*. 2002)

	EPSPs		IPSPs	
		Width at half‐		Width at half‐
	Rise time (ms)	amplitude (ms)	Rise time (ms)	amplitude (ms)
Interneurons	0.9 ± 0.3	7.2 ± 4.1	2.9 ± 0.5	10.1 ± 1.7
Excitatory cells	2.4 ± 1.1	19.4 ± 13.2	4.1 ± 0.6	20.3 ± 6.4

### Shunting inhibition and short‐interval intracortical inhibition and intracortical facilitation

Short‐interval intracortical inhibition (SICI) and intracortical facilitation (ICF) are classical paradigms in the TMS literature to investigate intracortical excitability (Kujirai *et al*. 1993). In this paradigm a conditioning subthreshold TMS pulse S1 is followed by a suprathreshold pulse S2 at different time intervals. The effect of S1 on the MEP evoked by S2 depends on the time interval between them; for the first 6 ms, S1 induces inhibition switching to excitation from 7 ms up to about 30 ms. The time course of SICI and ICF does not resemble that expected from the duration of a simple hyperpolarizing GABA_A_‐ergic IPSP, which should be purely inhibitory and last for 20 ms or more. It has been proposed that SICI/ICF represents an initial IPSP that is cut short by subsequent arrival of an EPSP (e.g. (Hanajima *et al*. 1998).

When a suprathreshold TMS pulse is applied to motor cortex, corticospinal neurons receive a rapid series of excitatory synaptic inputs that lead to repetitive high‐frequency waves of activity in the corticospinal tract. These are termed I‐waves because they are due to indirect (i.e. synaptic) activation of the pyramidal neurons. In order to produce SICI it is assumed GABAergic inputs have a low threshold and can be activated by subthreshold pulses. A puzzle about SICI is that it suppresses later I‐wave inputs much more than early inputs, no matter what the interval between S1 and S2. One possible reason is that the later inputs are located more distally than the GABAergic synapses and therefore are suppressed ‘on‐the‐line’ to the soma, whereas early inputs target more proximal locations. Such interactions have been modelled recently using a simplified fully feed‐forward arrangement of neural interactions without lateral connections or loops, based on a broad distribution of conduction delays of synaptic inputs arriving clustered at different parts of layer 5 cells’ dendritic trees (Rusu *et al*. 2014).

We would like to propose here that an additional explanation for the time course of SICI and ICF involves shunting inhibition and in some circumstances depolarizing GABAergic PSPs. Activation of the GABA synapse via a low‐threshold interneuron (Davey *et al*. 1994; Ilic *et al*. 2002) stimulated by S1 will lead to a dendritic GABA‐induced hyperpolarization with a superimposed short‐lasting initial period of shunting inhibition. The initial SICI that is observed at rest, and which is often very powerful, resembles the time course of shunting whereas the later facilitation is due either to summation of S2‐induced EPSPs or to paradoxical GABA‐induced membrane depolarization. Benzodiazepines increase SICI and decrease ICF. This dual effect could be because they increase channel opening times, prolonging shunting inhibition at short SICI intervals, and increase the effectiveness of longer lasting hyperpolarizing inhibition that suppresses ICF (Ziemann *et al*. 1996; Ilic *et al*. 2002; Di Lazzaro *et al*. 2005).

Voluntary activity reduces SICI and ICF. Indeed, Hanajima *et al*. (1998) found that ICF was absent when they tested intracortical inhibition during voluntary activity; they also noted that inhibition could persist for up to 20 ms consistent with the duration of a typical GABA_A_‐ergic hyperpolarizing IPSP. One explanation of these effects is that voluntary activity reduces membrane resistance and hence will reduce the effectiveness of both S1 and S2.

One feature of SICI supports the importance of a shunting contribution to the initial period of inhibition. If the intensity of S1 (the stimulus that provokes GABAergic inhibition) is raised it will begin to recruit excitatory inputs to pyramidal neurons. A complex experiment involving the interaction of SICI with short‐interval intracortical facilitation (SICF; or I‐wave interaction) suggests that any early excitatory inputs activated by a higher intensity S1 are quashed by inhibition but that later ones are not (Peurala *et al*. 2008).

A possible objection to a role of shunting inhibition in SICI is that SICI seems to target preferentially the late I‐wave excitatory inputs to the pyramidal neuron. If S1 were to initiate immediate inhibition of the neuron, then short‐duration shunting inhibition will be over by the time the late I‐wave inputs arrive. Thus it could play no role in their suppression. However, rather surprisingly perhaps, we do not know when inhibition begins after S1. Since activation of at least one interneuron is involved, it is possible that inhibition starts only shortly before the late I‐wave inputs arrive. In this case, a shunting effect could readily be an important contributor to SICI. It is difficult to prove the involvement of shunting inhibition in SICI in human brain. This is because its effect depends strongly on the relative location of synaptic inputs, which is unknown in the human experiments. In addition it is not possible to distinguish between strong shunting inhibition and powerful conventional hyperpolarizing inhibition by indirect measures. Such questions are much more suited to the newer animal models of the effects of TMS and tDCS that are being developed.

### Alternative accounts

Our discussion offers only a highly simplified account of the possible contribution of conductance changes to the effect of non‐invasive brain stimulation. We have considered only the site of stimulation and envisaged that the changes are evenly spread over a homogeneous network. However, in the real brain this is never likely to be the case. For example, tonic contraction will alter the spread of stimulation‐induced effects to interconnected network nodes, which for the motor system would include predominantly premotor cortex, putamen, cerebellum, spinal anterior horn and others (Andersen *et al*. 2014). Thus there will be widespread effects on brain activity far from the stimulated sites. Activation will also change the oscillatory activity of neural networks. This changes the temporal pattern of synaptic activity within the cortex adding a further dimension to the notion of activity‐driven conductance effects. Modelling work suggests that the effects of tDCS are not spatially homogeneous and that there may be a ‘speckled’ polarization where some populations are affected more strongly than others. The interaction with ongoing activity will therefore be very difficult to predict. Finally, as we have noted above for shunting inhibition, it is very difficult to prove or investigate the magnitude of these effects in the human setting. Data from tDCS and TMS in animal preparations are needed to fill the gaps in our knowledge and improve our understanding of the mechanisms of these useful, but puzzling, methods of interacting with the brain.

## Implications and conclusions

Measuring cortical excitability with TMS to the motor cortex and quantifying MEP amplitudes, SICI, ICF and recruitment curves has provided a unique framework for understanding concepts as well as parameters needed to induce rTMS as well as tDCS after‐effects. This framework depends mainly on data obtained in the resting motor cortex. Voluntary activation or focused attention modulates or even reverses these effects. By highlighting changes in membrane parameters that occur during activation, we show how TS effects may be linked with membrane impedance changes and shunting inhibition, a mechanism that has so far not been considered in TS models. Incorporating this information into existing anatomical and physiological models of TMS and tDCS may widen concepts and explain so far seemingly conflicting results.

## Additional information

### Competing interests

None of the authors has any conflicts of interests on the submission form.

### Author contributions

Both authors have approved the final version of the manuscript and agree to be accountable for all aspects of the work. All persons designated as authors qualify for authorship, and all those who qualify for authorship are listed.

### Funding

Walter Paulus was supported by the Deutsche Forschungsgemeinschaft, SPP 1665.
